# Microbiota-mediated modulation of radiosensitivity: mechanisms and therapeutic prospects of oral and gut microbiota, metabolites, and probiotics

**DOI:** 10.3389/fmicb.2025.1689735

**Published:** 2025-12-10

**Authors:** Baixue Wu, Anqi Zhao, Wenlu Chen, Yuying Zhou, Wenbo Zhang, Yiwen Zhang, Qing Hou, Ningning Yao, Shuangping Zhang, Jianchun Duan, Ning Li, Jianzhong Cao

**Affiliations:** 1 Cancer Hospital Affiliated to Shanxi Medical University, Taiyuan, China; 2 Department of Radiotherapy, Shanxi Province Cancer Hospital, Shanxi Hospital Affiliated to Cancer Hospital, Chinese Academy of Medical Sciences, Cancer Hospital Affiliated to Shanxi Medical University, Taiyuan, China; 3 CAMS Key Laboratory of Translational Research on Lung Cancer, State Key Laboratory of Molecular Oncology, Department of Medical Oncology, National Cancer Center/National Clinical Research Center for Cancer/Cancer Hospital, Chinese Academy of Medical Sciences and Peking Union Medical College, Beijing, China; 4 Respiratory Department, Shanxi Province Cancer Hospital, Shanxi Hospital Affiliated to Cancer Hospital, Chinese Academy of Medical Sciences, Cancer Hospital Affiliated to Shanxi Medical University, Taiyuan, Shanxi, China

**Keywords:** microbiota, radiosensitivity, short-chain fatty acids (SCFAs), probiotics, radiotherapy, tumor microenvironment

## Abstract

Radiotherapy is a cornerstone of comprehensive cancer treatment, yet its efficacy and toxicity exhibit considerable interindividual variation. Recent evidence highlights the microbiome—the collective genomes and metabolic products of symbiotic microorganisms in a specific environment—as a key bidirectional regulator of radiosensitivity. Radiotherapy can disrupt microbial community structure, while the microbiome and its metabolites profoundly influence tumor cell radiosensitivity and normal tissue radiotolerance by modulating DNA damage repair, immune responses, metabolic reprogramming, and tumor microenvironment (TME) remodeling. This review systematically examines the mechanisms and recent advances in understanding how oral and gut microbiota, their key metabolites (e.g., short-chain fatty acids, SCFAs), and probiotics modulate radiosensitivity. By establishing a framework centered on “mechanism axis—evidence stratification—clinical translation,” this paper aims to provide a theoretical foundation and identify potential targets for microbiome-based strategies to enhance radiosensitivity and protect normal tissues during radiotherapy.

## 1 Introduction

Radiotherapy (RT) is a critical modality in cancer treatment, administered to approximately 70% of patients during their course (Luo et al., 2005). It utilizes ionizing radiation to induce DNA damage directly or indirectly (via free radicals generated from water radiolysis), thereby suppressing or eliminating tumors to alleviate symptoms, prolong survival, and improve quality of life. However, its clinical application faces two major challenges: first, treatment efficacy varies significantly due to factors such as genetic background, age, and comorbidities (Manem and Taghizadeh-Hesary, 2024), hindering the implementation of precision radiotherapy; second, radiation-induced damage to adjacent normal tissues (e.g., oral mucositis, enteritis) often limits dose escalation, reducing patient tolerance and compromising treatment outcomes (Voshart et al., 2021).

Recent advances in human microbiome research offer novel insights into these challenges. The human body hosts a vast community of symbiotic microorganisms, whose collective genetic material (the microbiome) is intricately linked to host health (Liu et al., 2022; Kerr, 2015). These microbes participate in immune activation, metabolic regulation, and barrier defense (Khan et al., 2024), and are increasingly recognized for their roles in cancer therapy. Studies indicate that radiotherapy can induce dysbiosis in the oral and gut microbiota, such as a decline in beneficial bacteria and an increase in potentially pathogenic bacteria, which correlates with both side effects and variations in treatment efficacy (Yi et al., 2019). Notably, the microbiome actively modulates tumor radiosensitivity through metabolites such as SCFAs and interactions with the host immune system (Li et al., 2022).

This review centers on the theme “Mechanistic axes of microbiome regulation of radiosensitivity — evidence stratification — clinical translation.” We systematically summarize the mechanisms and research progress regarding the role of the microbiome (focusing on oral and gut microbiota), key metabolites (such as SCFAs), and probiotics in regulating radiosensitivity, aiming to provide a foundation for microbiome-based strategies to improve radiotherapy outcomes.

## 2 Mechanistic axis of microbiome regulation of radiosensitivity

From the oral cavity to the rectum, the human body harbors a complex microbial ecosystem that coevolves with the host to maintain metabolic, immune, and endocrine homeostasis (Liu et al., 2022). Dietary components such as fiber are fermented by gut microbiota to produce various metabolites, including SCFAs (acetate, propionate, and butyrate) (Then et al., 2024; Gately, 2019). These microbial components and metabolites bidirectionally regulate tumor radiosensitivity and the radiotoxicity of normal tissues through four core pathways: DNA damage repair, immune activation, metabolic reprogramming, and TME remodeling, forming a multidimensional regulatory network.

### 2.1 DNA damage repair and replication fork protection pathways

Radiotherapy primarily acts by inducing DNA double-strand breaks (DSBs). Cellular efficiency in DSB repair and replication fork stability are key determinants of radiosensitivity (Mladenov et al., 2013). The microbiome influences tumor cell response by modulating repair pathways such as non-homologous end-joining (NHEJ) and homologous recombination (HR).

#### 2.1.1 Regulation of NHEJ

**Figure 1:**
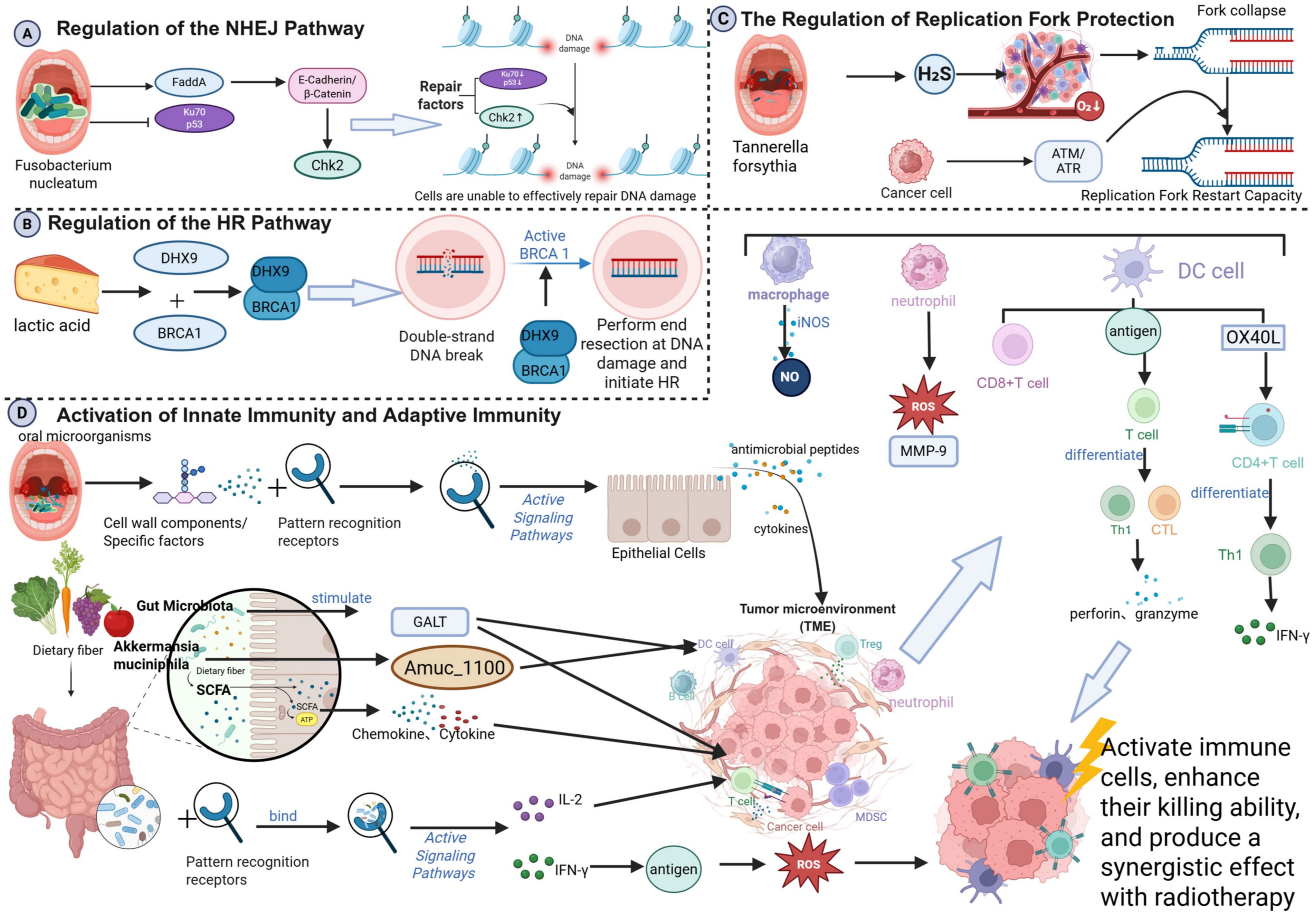
**(A)** Regulation of the NHEJ pathway; **(B)** regulation of the HR pathway; **(C)** regulation of the HR pathway; **(D)** activation of innate immunity and adaptive immunity; (created in BioRender. Cao, J. 2025 https://BioRender.com/dzmqbn3).

Oral *Fusobacterium nucleatum* can induce DNA damage via virulence proteins (e.g., FadA), which activate E-cadherin/*β*-catenin signaling, upregulate Chk2, and promote genomic instability (Guo et al., 2020). Additionally, *Clostridium difficile* infection downregulates Ku70, a key NHEJ protein, and wild-type p53, impairing DNA repair and potentially leading to radiation resistance through accumulated genomic instability (Geng et al., 2020; Jiadi and Songqing, 2014) (Figure 1A).

#### 2.1.2 Regulation of HR

HR is a high-fidelity DSB repair pathway active in S/G2 phase, reliant on protein complexes such as BRCA1, BRCA2, and RAD51 (Prakash et al., 2015)—defects in HR increase radiosensitivityy (Jiabei et al., 2018). Gut microbiota-derived lactate can promote DHX9-BRCA1 interaction, facilitating HR initiation and conferring radioresistance (Xiao et al., 2025). DHX9 knockdown enhances radiosensitivity in hepatocellular carcinoma (Chen et al., 2022) (Figure 1B).

#### 2.1.3 Regulation fork protection

Radiation can stall or collapse replication forks, leading to cell death if not stabilized (Nickoloff, 2022; Nickoloff et al., 2023). Oral *Fusobacterium nucleatum* exacerbates TME hypoxia via hydrogen sulfide production (Zhao et al., 2024), inducing fork collapse. Tumor cells may counteract this by activating the ATM/ATR pathways to restart forks, potentially inducing resistance (Zhao et al., 2024) (Figure 1C).

### 2.2 Immune activation and immune checkpoint regulation pathways

Radiotherapy induces immunogenic cell death (ICD) (Kaur and Asea, 2012), releasing damage-associated molecular patterns (DAMPs, e.g., ATP and HMGB1) and tumor antigens that activate dendritic cells (DCs), promote antigen presentation, and initiate T-cell responses (Herrera et al., 2017; Hernandez et al., 2016)^.^

#### 2.2.1 Innate immunity and adaptive immunity

Beneficial oral microbes bind pattern recognition receptors on the epithelial cells, triggering antimicrobial peptides and cytokines secretion, and recruiting macrophages and neutrophils to tumor sites (Chung et al., 2010; Lamont et al., 2023). These cells release cytotoxic substances, such as reactive oxygen species (ROS), nitric oxide (NO), and matrix metalloproteinase-9 (MMP-9), which directly kill tumor cells and enhance the radiation response. Specifically, neutrophils release ROS and MMP-9 to disrupt the tumor extracellular matrix (ECM) and induce DNA damage (Huang et al., 2024; Gibellini et al., 2023); macrophages produce NO via inducible nitric oxide synthase (iNOS) to directly kill tumor cells (Szabo, 2016). Oral microbes also enhance DC antigen presentation, activating cytotoxic T lymphocytes (CTLs) and Th1 cells (Ebersole et al., 2000)^,^ which release perforin and granzyme to amplify radiation-induced DNA damage (Yin et al., 2024; Park et al., 2020). Specific oral microbes upregulate OX40L on DCs, promoting Th1 differentiation and IFN-*γ* secretion (Ito et al., 2005). SCFAs modulate chemokine/cytokine expression, attracting and activating T cells and NK cells (Adelinik et al., 2023; Campbell and Decker, 2017). Gut microbiota stimulate gut-associated lymphoid tissue (GALT), activating DCs and T cells (Yu et al., 2024). Probiotics (e.g., *Bifidobacterium* and *Lactobacillus*) bind immune cell receptors to promote cytokine secretion (e.g., IL-2, IFN-γ) (Bermudez-Brito et al., 2012; Hamada et al., 2018)^,^ enhancing antitumor cytotoxicity and radiosensitivity (Kaur and Asea, 2012). *Akkermansia muciniphila* enhances DC function via Amuc-1100, thereby boosting CD8 + T cell infiltration and improving melanoma radiotherapy outcomes (Chang et al., 2019) (Figure 1D).

#### 2.2.2 Immune checkpoint regulation

**Figure 2:**
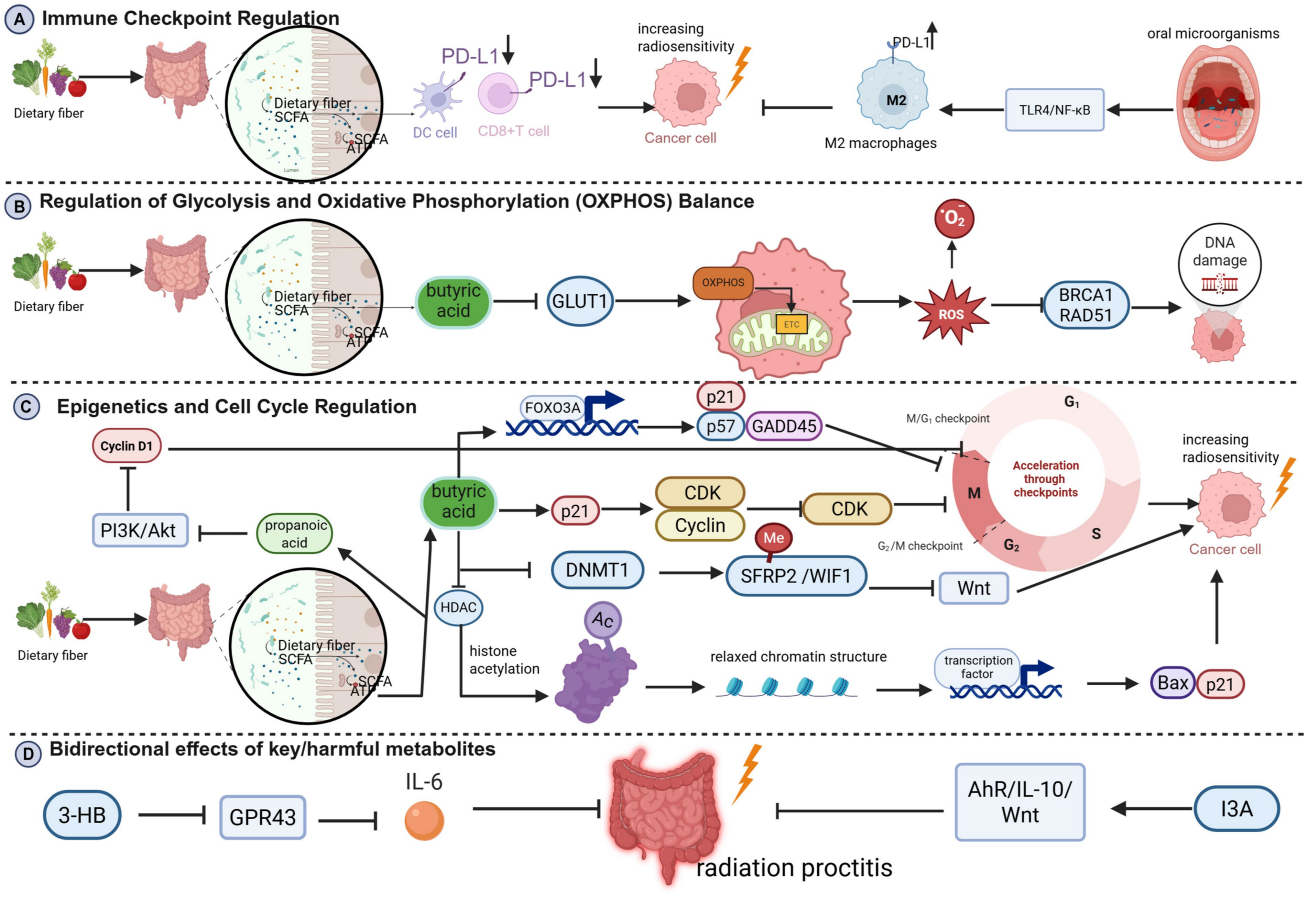
**(A)** Immune checkpoint regulation; **(B)** regulation of glycolysis and oxidative phosphorylation (OXPHOS) balance; **(C)** epigenetics and cell cycle regulation; **(D)** bidirectional effects of key/harmful metabolites (Created in BioRender. Cao, J. (2026) https://BioRender.com/y4y0mn8).

Tumor cells often evade immunity via PD-L1 upregulation. Gut microbiota and SCFAs can reduce PD-L1 expression by activating DCs and CD8+ T cells (Ding et al., 2018; Routy et al., 2024), thereby alleviating immunosuppression (Dyer et al., 2019). Conversely, *Fusobacterium nucleatum* activates TLR4/NF-κB pathway, promotes M2 macrophage polarization, upregulates PD-L1, and induces immunosuppression, reducing radiosensitivity (Zhang et al., 2024; Wanyi et al., 2023) (Figure 2A).

### 2.3 Metabolic reprogramming pathways

Tumor cells sustain proliferation through metabolic reprogramming, such as enhanced glycolysis. Microbiome metabolites, particularly SCFAs, can regulate tumor cell energy metabolism and influence radiosensitivity.

#### 2.3.1 Glycolysis-OXPHOS balance

SCFAs, especially butyrate, shift tumor metabolism from glycolysis to oxidative phosphorylation (OXPHOS) by downregulating GLUT1 and activating mitochondrial respiration (Geng et al., 2021; Vander Heiden et al., 2009). Enhanced OXPHOS increases electron leakage in the mitochondrial electron transport chain (ETC), resulting in the generation of superoxide anion (O₂^−^) and reactive oxygen species (ROS) (Liskova et al., 2021). ROS accumulation synergizes with radiation-induced free radicals, inhibits DNA repair proteins (e.g., BRCA1, Rad51), and disrupts redox homeostasis, increasing oxidative damage and radiosensitivity (Bhattacharya and Asaithamby, 2017; Laurini et al., 2020; Kobylinska et al., 2019) (Figure 2B).

#### 2.3.2 Epigenetics and cell cycle regulation

SCFAs induce cell arrest at radiation-sensitive phases (e.g., G2/M) by modulating cyclin expression and activity. Butyrate upregulates p21, inhibiting CDK activity by binding to the cyclin-CDK complex and causing G0/G1 or G2/M arrest (Foglietta et al., 2014; Bose and Sarma, 1975; Sandor et al., 2000). G2/M arrest enhances radiation-induced apoptosis and suppresses DNA repair (e.g., Rad51 downregulation) (Sahin et al., 2019). Butyrate also potentiates FOXO3A (Forkhead box class O3A) function, regulating p21, p57, and GADD45 (Growth Arrest and DNA Damage-Induced Protein 45) to induce arrest (Lu et al., 2024). SCFAs inhibit DNA methyltransferases (e.g., DNMT1), leading to demethylation and re-expression of tumor suppressor genes (e.g., SFRP2, WIF1), which in turn inhibit Wnt signaling and tumor growth (Feitelson et al., 2023). As HDAC inhibitors, SCFAs increase histone acetylation, loosening chromatin and activating pro-apoptotic (e.g., Bax) and cell cycle arrest genes (e.g., p21) (Han et al., 2018; Sun et al., 2018; Gong et al., 2023). Propionate inhibits PI3K/Akt, reduces cyclin D1, and induces G1 arrest, also sensitizing cells to radiation (Feitelson et al., 2023; Ye et al., 2022; Gong et al., 2024) (Figure 2C).

#### 2.3.3 Bidirectional effects of metabolites

Radiotherapy alters microbial metabolism, leading to the production of ROS, hydrogen sulfide, and lactic acid, among others. At low concentrations, hydrogen sulfide activates the MAPK signaling pathway, thereby promoting the proliferation and survival of tumor cells. For example, studies have shown that hydrogen sulfide can enhance the proliferative capacity of tumor cells by activating signaling pathways, such as ERK1/2 and p38 MAPK (Gao et al., 2024). However, in tumor cells, high concentrations of hydrogen sulfide can induce apoptosis. It can trigger tumor cell apoptosis through multiple mechanisms, including the activation of the unfolded protein response (UPR), promotion of cell cycle arrest, and suppression of anti-apoptotic protein expression (Ma et al., 2023).

Radiation therapy-induced alterations in the oral environment (such as inflammation and hypoxia) can promote the production of harmful metabolites (e.g., candidalysin, acetaldehyde) by certain oral microorganisms (e.g., *Candida albicans*). These changes may also influence radiosensitivity by inducing chronic inflammation or DNA damage, thereby modifying the oral tumor microenvironment (Li et al., 2024).

Gut microbiota-derived 3-hydroxybutyrate (3-HB) can suppress radiation therapy-induced rectal mucosal inflammation and improve radiation proctopathy by blocking GPR43-mediated IL-6 signaling pathways (Ge et al., 2024). Meanwhile, the tryptophan metabolite indole-3-acetic acid (I3A) protects the gut from radiation damage by activating the AhR/IL-10/Wnt signaling pathway. In gastric cancer, it also suppresses PD-L1 expression, thereby enhancing radiosensitivity (Xie et al., 2024) (Figure 2D).

### 2.4 Tumor microenvironment (TME) remodeling pathways

The TME, comprising stroma, vasculature, and immune cells, critically influences radiotherapy response through angiogenesis, extracellular matrix (ECM) structure, and hypoxia.

#### 2.4.1 Angiogenesis regulation

Gut microbes can secrete factors affecting VEGF (Fakruddin et al., 2024), inhibiting angiogenesis and inducing hypoxia (Yu and Cui, 2018). *Porphyromonas gingivalis* promotes angiogenesis via VEGF, exacerbating hypoxia, and reducing radiosensitivity (ChenXi et al., 2024; Yibo et al., 2025). Butyrate downregulates VEGF by inhibiting the activity of the hypoxia-inducible factor HIF-1α, reducing angiogenesis and inducing hypoxia (Hou et al., 2024).

**Figure 3:**
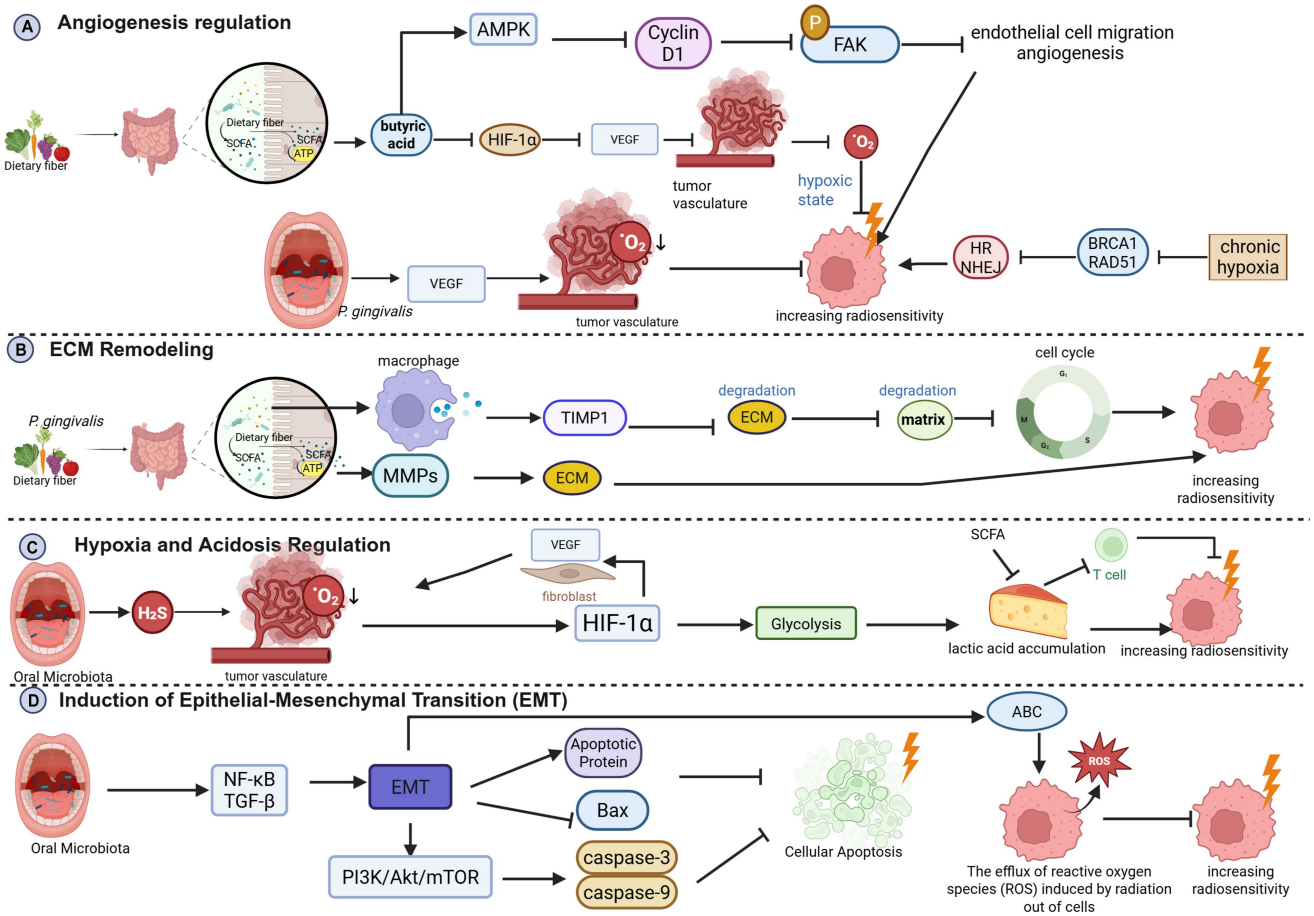
**(A)** Angiogenesis regulation; **(B)** ECM remodeling; **(C)** hypoxia and acidification regulation; **(D)** induction of epithelial-mesenchymal transition (EMT) (Created in BioRender. Cao, J. (2026) https://BioRender.com/bisrh40).

Chronic hypoxia downregulates DNA repair proteins (e.g., BRCA1, Rad51), inhibiting HR and NHEJ, increasing radiosensitivity (Berge et al., 2001; Li and Greenberg, 2012; van de Kamp et al., 2021; Gutschner and Diederichs, 2012; Gorodetska et al., 2019). SCFAs also directly inhibit endothelial cell proliferation and migration via AMPK activation and suppression of FAK phosphorylation (Mathew et al., 2019; Yoo et al., 2021) (Figure 3A).

#### 2.4.2 ECM remodeling

Gut microbiota influence ECM stiffness by regulating macrophage TIMP1 secretion, reducing degradation (Peng et al., 2024). Altered ECM affects tumor cell adhesion, migration, and proliferation, thereby increasing radiation susceptibility (Shiao and Coussens, 2010). SCFAs inhibit matrix metalloproteinase (MMP) activity, maintaining ECM stability and synergizing with radiotherapy (Gomes et al., 2023) (Figure 3B).

#### 2.4.3 Hypoxia and acidification

Oral *Fusobacterium nucleatum* exacerbates TME hypoxia via H_2_S (Hsieh et al., 2022), activating HIF-1α, promoting glycolysis and lactic acid accumulation (Gomes et al., 2023). Lactate acidifies the TME, suppressing the cytotoxicity of immune cells (T cells, NK cells, and DCs) and creating an immunosuppressive cycle (Pérez-Tomás and Pérez-Guillén, 2020). Lactate also stabilizes HIF-1α, promoting VEGF expression and angiogenesis (Missiaen et al., 2023). SCFAs reduce lactate production by inhibiting tumor glycolysis, alleviating acidification, restoring immune function, and enhancing radiosensitivity (Rah et al., 2025) (Figure 3C).

#### 2.4.4 Epithelial-mesenchymal transition (EMT)

Oral pathogens like *P. gingivalis* and *F. nucleatum* can disseminate, promoting tumor invasiveness and EMT via NF-κB and TGF-*β* pathways (Palkovsky et al., 2025; Kerr, 2015). EMT upregulates anti-apoptotic proteins (Bcl-2, Bcl-xL), downregulates Bax, inhibits caspase activity, and upregulates ABC transporters, effluxing drugs and ROS, leading to radioresistance and increased invasiveness (Zhang and Weinberg, 2018; Peyre et al., 2021; Yarahmadi and Afkhami, 2023; Lee et al., 2017; Yarahmadi and Afkhami, 2023). *Clostridium difficile* increases the metastatic burden and impairs the efficacy of radiotherapy, while its targeted elimination enhances radiosensitivity (Kerr, 2015; Yarahmadi and Afkhami, 2023) (Figure 3D).

## 3 Evidence stratification for microbiome-related factors and interventions

The roles of oral and gut microbiota, their key metabolites, and probiotics in modulating radiosensitivity are context-dependent, influenced by factors such as tumor type, radiotherapy regimen, and host immune status, with varying levels of supporting evidence. This section stratifies the evidence and analyzes the effects of core entities—including probiotics, short-chain fatty acids (SCFAs), *Akkermansia muciniphila*, and lactic acid bacteria/bifidobacteria—under different conditions to inform future precision interventions (see Supplementary Table S1 for details).

### 3.1 Effects of probiotics on radiosensitivity

Probiotics are live, non-pathogenic microorganisms that confer health benefits when administered in sufficient amounts (Gong and Li, 2025). Traditional probiotics include *Lactobacillus* and *Bifidobacterium*, while Next-Generation Probiotics (NGPs) are specific symbiotic bacteria identified from the human gut microbiome through high-throughput sequencing, often with broader physiological functions (Al-Fakhrany and Elekhnawy, 2024).

#### 3.1.1 Examples of new-generation probiotic candidate

*Akkermansia muciniphila* is a Gram-negative bacterium colonizing the intestinal mucosal layer. It utilizes mucin as a source of carbon and nitrogen, contributing to the maintenance of mucus layer thickness and intestinal barrier integrity. *Akkermansia muciniphila* or its outer membrane protein Amuc-1100 can alleviate colitis symptoms, modulate immune responses, reduce intestinal inflammation levels, and prevent colitis-associated colorectal cancer. Notably, *Akkermansia muciniphila* is significantly enriched in cancer patients responsive to PD-1 immunotherapy, suggesting it may enhance the efficacy of anti-PD-1 and other immunotherapies.

*Faecalibacterium prausnitzii* constitutes approximately 5% of the gastrointestinal microbiome and is one of the primary butyrate producers. It promotes the synthesis of mucins and tight junction proteins, repairs damaged intestinal mucosa, and regulates mucus secretion, playing a crucial role in maintaining gut health. Research further indicates that its abundance in the gut correlates with long-term survival in cancer patients. During melanoma metastasis, blood levels of regulatory T cells and specific pro-inflammatory cytokines negatively correlate with the abundance of *Faecalibacterium prausnitzii* in the gut, positioning it as a potential key therapeutic target and prognostic biomarker for melanoma patients (Chang et al., 2019).

#### 3.1.2 Mechanism by which probiotics enhance radiation sensitivity

##### 3.1.2.1 Restoring gut microbiota imbalance after radiation therapy

###### 3.1.2.1.1 Competitive colonization

Probiotics inhibit the growth of pathogens (such as *Escherichia coli* and Clostridium species) through displacement effects and nutrient competition, reducing the production of harmful metabolites (such as lipopolysaccharides and hydrogen sulfide). Simultaneously, they promote the proliferation of beneficial bacteria (such as butyrate-producing bacteria), thereby optimizing the intestinal metabolic environment (Anee et al., 2021; Patel and DuPont, 2015).

###### 3.1.2.1.2 Barrier repair

Probiotics promote the secretion of mucin MUC-2 and antimicrobial peptides (such as defensins) by the intestinal mucosa, repairing radiation-damaged intestinal barriers and reducing inflammatory responses triggered by endotoxin translocation (Wu et al., 2022).

###### 3.1.2.1.3 Clinical evidence supports

A study on colorectal cancer patients demonstrated that probiotic supplementation significantly reduces chemotherapy-induced gastrointestinal complications, particularly diarrhea. Furthermore, probiotics restore gut bacterial diversity post-chemotherapy, alter the composition of the microbiota, and increase short-chain fatty acid (SCFA) production (Huang et al., 2023).

##### 3.1.2.2 Enhancing radiation therapy-induced immune activation

Against the backdrop of radiation therapy-induced immunosuppression, probiotics can amplify the immunogenic cell death (ICD) effect of radiotherapy through innate immune modulation, such as activating dendritic cells and promoting Th1-type immune responses.

###### 3.1.2.2.1 Reactivation of immune cells

Activation of natural killer (NK) cells and cytotoxic T lymphocytes (CTLs) enhances the ability to eliminate residual tumor cells following radiotherapy (Laurent et al., 2023).

###### 3.1.2.2.2 Cytokine rebalancing

Downregulates pro-inflammatory factors (such as IL-6 and TNF-*α*, which are often excessively induced by radiotherapy) and upregulates anti-tumor factors (such as IFN-*γ* and IL-12), thereby reversing the immunosuppressive microenvironment (Ni et al., 2023; Zhao et al., 2021).

##### 3.1.2.3 Synergistic effects of probiotic metabolic products

Probiotics ferment dietary fibers that the hosts cannot digest (such as pectin and inulin), producing SCFAs. This process relies on key bacterial enzymes including glycosidase and phosphotransferase (Sunkata et al., 2014).

###### 3.1.2.3.1 Key roles of SCFAs

Butyrate and propionate produced by probiotic metabolism enhance immune cell function. Butyrate promotes CD8+ T cell proliferation and memory T cell formation by activating the free fatty acid receptor 3 (FFAR3). Propionate alleviates intestinal mucosal cell apoptosis and mitigates radiation enteritis by inhibiting histone deacetylase (HDAC) (Hsieh et al., 2022).

###### 3.1.2.3.2 Improving radiotherapy tolerance

SCFAs enhance intestinal barrier function, reduce bacterial translocation and endotoxin release following radiotherapy, thereby stabilizing the body’s condition and increasing tumor cells’ sensitivity to radiotherapy (Ye et al., 2022). Additionally, SCFAs provide energy to intestinal cells, maintaining their normal function, and influence tumor cell gene expression by regulating HDAC activity, making them more sensitive to radiotherapy (Kim et al., 2016).

##### 3.1.2.4 Directly regulate tumor cell biological behavior

###### 3.1.2.4.1 Inducing tumor cell apoptosis

For example, cell wall components of Bifidobacteria (such as peptidoglycan and lipopolysaccharide) activate TLR2 and NLRs, triggering NF-κB and MAPK signaling pathways to regulate Bcl-2/Bax expression (Chapot-Chartier and Kulakauskas, 2014); Certain bifidobacteria (such as *Bifidobacterium longum* and *Bifidobacterium breve*) can produce butyrate through the fermentation of dietary fiber and other substrates (Duda-Chodak et al., 2015). Research indicates that butyrate promotes apoptosis by enhancing the activity of isocitrate dehydrogenase 1 (IDH1) and the pyruvate dehydrogenase complex (PDH), thereby increasing α-ketoglutarate production and upregulating the Bax/Bcl-2 ratio (Zhang et al., 2024). By downregulating Bcl-2 expression and upregulating the pro-apoptotic protein Bax, the mitochondrial apoptosis pathway is activated. When combined with radiotherapy, caspase-3/9 activity (cysteine proteases closely associated with apoptosis) increases significantly, leading to enhanced apoptosis rates (Dehghani et al., 2021). Probiotics synergistically enhance apoptosis signaling with radiotherapy, thereby improving therapeutic efficacy.

###### 3.1.2.4.2 Induction of cell cycle arrest

**Figure 4:**
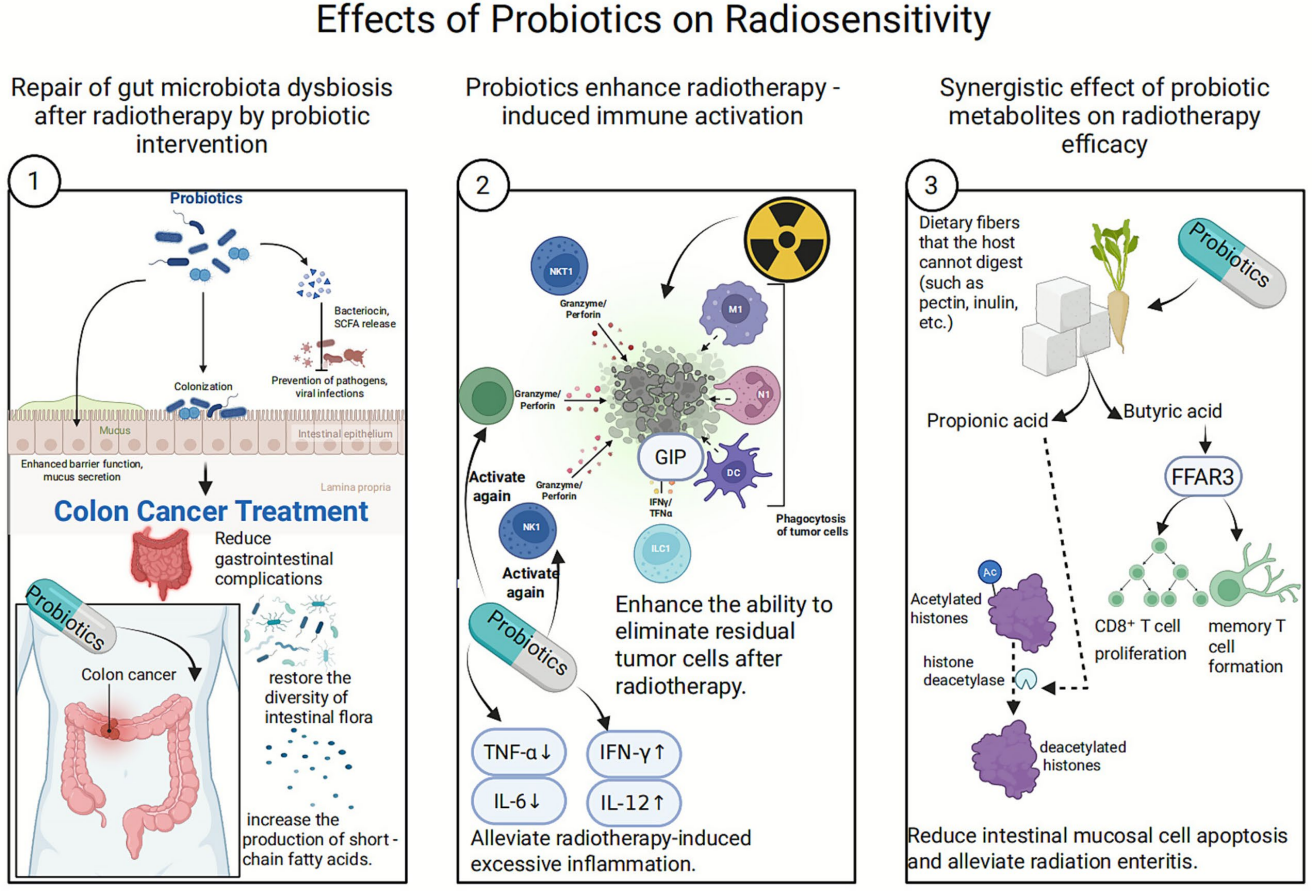
Schematic diagram of the pleiotropic mechanism by which probiotic.

Probiotics induce tumor cell arrest at the G2/M phase—a stage sensitive to radiotherapy—by regulating p21 and Cyclin B1/CDK1 (Yu et al., 2017). This arrest prolongs the exposure time to DNA damage induced by radiotherapy. Concurrently, probiotics inhibit proliferation signals through the AMPK/mTOR or Wnt pathways, thereby reducing tumor cell divisio (Palkovsky et al., 2025) and rendering them more susceptible to radiation-induced cell death (Figure 4).

### 3.2 Short-chain fatty acids (SCFAs)—taking butyrate as an example

Butyrate significantly enhances radiation-induced cell death in three-dimensional organoids derived from colorectal cancer patients, primarily by activating FOXO3A, which in turn inhibits S-phase cell proliferation. However, in normal colon organoids, butyrate did not enhance radiation lethality; instead, it protected mucosal regeneration, indicating that its radiosensitizing effect is tissue-specific. *In vitro* experiments revealed radiosensitization occurred in only 3 out of 8 organoids examined and depended on FOXO3A expression levels, suggesting molecular profiling of tumors should precede its application (Park et al., 2020). On the other hand, butyrate activates GPR43 (FFAR2) to promote the proliferation of colonic mucosal T regulatory cells (Tregs), thereby helping to suppress experimental colitis. However, during specific windows of radiotherapy, excessive butyrate may lead to Treg over-expansion, suppressing antitumor immunity and altering PD-L1 dynamics, thereby diminishing the efficacy of radiotherapy combined with immune checkpoint inhibitors (Cook and Sellin, 1998). Animal models and *in vitro* cell experiments suggest that dosage and timing have a significant influence on outcomes. Excessive doses or improper administration timing may shift butyrate’s immunomodulatory effects toward immunosuppression. Therefore, precise calibration of dosage and temporal windows is crucial for achieving sensitization while avoiding adverse immunomodulation.

### 3.3 *Akkermansia muciniphila*—a double-edged sword effect

In models of radiation-induced intestinal injury, *Akkermansia muciniphila* exhibits a dual-edged effect. When administered orally to mice with intact, healthy mucosal layers following whole-body abdominal irradiation (single or fractionated doses at relatively low levels) using the laboratory standard strain ATCC 8483 (or equivalent low-dose strains), it significantly reduces intestinal injury scores and improves survival rates. Its metabolite, propionic acid, enhances histone acetylation via the GPR43 receptor, promoting the expression of tight junction proteins, including occludin, ZO-1, and mucin MUC2, to reinforce the intestinal barrier. Concurrently, it suppresses the TLR2/MyD88/NF-κB inflammatory pathway, leading to reduced inflammatory mediators and mitigated radiation injury (He et al., 2023). However, another study revealed that when *A. muciniphila* increased in the intestines of mice after radiation exposure, supplementation with this bacterium actually exacerbated mucosal thinning and inflammatory macrophage infiltration, leading to higher mortality rates. Mechanistically, *A. muciniphila* compromises the mucus barrier by upregulating genes involved in mucin metabolism and directly utilizing mucins as an energy source. Concurrently, it activates inflammatory macrophages and promotes the secretion of inflammatory cytokines (such as TNF-*α* and IL-6), thereby suppressing intestinal stem cell density and goblet cell differentiation, further impairing mucosal repair capacity (Wang et al., 2025). The experimental settings differed between the two studies: the former employed whole-body abdominal irradiation with a low-dose strain. At the same time, the latter employed localized small-intestinal irradiation with a high-dose strain, yielding opposing conclusions. Strains from different sources (e.g., ATCC 8483 versus clinical isolates) exhibit differences in genotype and metabolite profiles. This leads to protective effects in healthy hosts with intact mucus, but may shift to pathogenicity in environments with compromised mucus or rapid mucus degradation following radiation exposure.

### 3.4 Ketone bodies—using 3-hydroxybutyric acid (3-HB) as an example

The protective effect of 3-hydroxybutyrate (3-HB) in radiation-induced proctitis primarily occurs through inhibiting GPR43-mediated IL-6 signaling. Radiation induces gut microbiota dysbiosis, particularly a decline in the abundance *Akkermansia muciniphila*, which reduces intestinal 3-HB production. Consequently, the antagonistic effect on GPR43 is lost, leading to IL-6 overexpression and exacerbated inflammation. Supplementing with 3-HB (or restoring *A. muciniphila*) can re-inhibit GPR43, decrease STAT3 phosphorylation levels, significantly reduce the inflammatory cytokine IL-6, and thereby improve IL-6 levels.-6 signaling mediated by GPR43. Radiation induces gut dysbiosis, notably by reducing the abundance of *Akkermansia muciniphila*, which in turn diminishes intestinal 3-hydroxybutyrate (3-HB) production. The loss of 3-HB antagonism against GPR43 results in excessive IL-6 expression and exacerbated inflammation. Supplementing with 3-HB (or restoring *A. muciniphila*) reinstates GPR43 inhibition, reduces STAT3 phosphorylation, and significantly lowers the inflammatory cytokine IL-6, thereby improving histological damage and clinical symptoms in radiation-induced proctitis. Relevant experiments, including mouse models of radiation-induced proctitis, *in vitro* studies with intestinal epithelial cells, and serum/fecal 3-HB measurements, consistently demonstrate the significant therapeutic effect of this metabolite on the disease.-HB concentrations in serum/feces demonstrated a significant disease-alleviating impact of this metabolite (Ge et al., 2024).

### 3.5 Tryptophan-derived metabolites—taking indole-3-carboxylic acid (I3A) as an example

Indole-3-carboxylic acid (I3A) protects the intestine from radiation damage by activating the AhR-carboxylic acid (I3A) protects the intestine from radiation damage by activating the AhR/IL-10/Wnt signaling pathway. As an endogenous ligand for AhR, I3A binds to AhR and upregulates IL-10 to create an anti-inflammatory microenvironment. Simultaneously, it activates the Wnt/*β*-catenin pathway to promote the proliferation and differentiation of intestinal stem cells, thereby enhancing the mucosal regeneration capacity. More importantly, I3A also increases the abundance of probiotics (such as *Lactobacillus*, *Bifidobacterium*, etc.), further strengthening the intestinal barrier function. Both mouse models of radiation-induced intestinal injury and intestinal organoid experiments demonstrated that I3A administration significantly improved histological scores, barrier integrity, and animal survival rates, while showing no radioprotective effects on tumor cells, indicating good selective safety (Xie et al., 2024).

### 3.6 Dietary fiber/antibiotics/phage therapy

Dietary fiber (such as inulin and pectin) significantly enhances short-chain fatty acid (SCFA) production by regulating the gut microbiota, thereby rapidly altering human gut bacterial diversity and increasing SCFA levels in feces (Then et al., 2024). In a mouse model of single-dose 10 Gy abdominal irradiation, drinking water containing 4% apple pectin reduced intestinal wall thickening, collagen deposition, and EMT marker expression, accompanied by elevated fecal SCFA levels. This suggests that fiber protects the intestine after radiation therapy through the gut microbiota-SCFA axis (Yang et al., 2017). The corresponding randomized controlled clinical trial in humans divided subjects into three groups: low fiber (≤10 g NSP/d), conventional fiber (control), and high fiber (≥18 g NSP/d). Results demonstrated significantly reduced gastrointestinal toxicity scores in the high-fiber group during and after radiotherapy (*p* = 0.011), with sustained improvement in gastrointestinal symptoms at the 1-year follow-up. This directly confirms that high-dose soluble dietary fiber can reduce the incidence of radiation-induced diarrhea (Wedlake et al., 2017). As demonstrated by the preceding discussion and experimental evidence, dietary fiber exerts multiple effects in slowing tumor growth and enhancing the efficacy of anticancer treatments by optimizing microbial metabolism, strengthening the intestinal barrier, and regulating the immune microenvironment. In patients with advanced pancreatic or colorectal cancer complicated by intestinal strictures, high-fiber foods (such as celery and coarse grains) may accumulate in the intestinal lumen due to inadequate digestion, potentially leading to intestinal obstruction or even perforation (Hassranah et al., 2022).

Vancomycin enhances the antitumor activity of radiotherapy by selectively eliminating Gram-positive bacteria in the gut, thereby improving the presentation of tumor-associated antigens to CD8+ T cells (Al-Qadami et al., 2023). Pre-treatment administration of antibiotics targeting *Porphyromonas gingivalis* (e.g., metronidazole) before oral radiotherapy reduces the incidence of bacteria-mediated oral mucositis and decreases the risk of radiotherapy interruption due to inflammation (Alterio et al., 2007). In contrast, broad-spectrum antibiotics such as cephalosporins and quinolones lack selectivity toward beneficial gut bacteria (e.g., bifidobacteria and SCFA-producing lactobacilli), leading to a significant decline in microbial diversity and reduced SCFA production. This subsequently weakens the intestinal barrier function and exacerbates the occurrence of radiation enteritis (Modi et al., 2014).

Following the administration of broad-spectrum antibiotics in patients undergoing stereotactic body radiation therapy (SBRT) for lung cancer, the abundance of SCFA-producing gut bacteria decreased, SCFA-mediated ROS accumulation effects weakened, tumor cell DNA repair capacity increased, and local recurrence rates consequently rose (Liskova et al., 2021; Xu et al., 2022). Long-term antibiotic use facilitates the dissemination of resistance genes, such as *β*-lactamases, among gut bacteria, thereby forming a reservoir of resistance genes. Following radiotherapy, patients with dysbiosis are more susceptible to refractory Clostridioides difficile infections, which further worsen clinical outcomes (Rochegüe et al., 2021). Overall, the selection and timing of antibiotics during radiotherapy critically influence tumor control, normal tissue preservation, and the risk of resistance, necessitating careful clinical deliberation.

Phage therapy is an anti-infective strategy that utilizes bacteriophage—bacteria-specific viruses—to lyse pathogenic bacteria directly. Due to its high specificity, self-replication capability, and ability to disrupt biofilms, it is regarded as a potential alternative to address the antibiotic resistance crisis. In recent years, clinical research has advanced rapidly: Phase I/II trials conducted in Belgium, the United States, Australia, and other countries have demonstrated significant safety and efficacy against multidrug-resistant bacteria such as *Staphylococcus aureus*, *Pseudomonas aeruginosa*, and *Acinetobacter baumannii* (Sawa et al., 2024); Meanwhile, several challenges remain: (1) The host specificity of phages necessitates customization for each patient or rapid screening for effective strains; (2) Production must comply with GMP standards, yet globally compliant facilities are scarce; (3) Immunogenicity and the generation of anti-phage antibodies may diminish efficacy, requiring management through fractionated dosing or immunosuppressive strategies (Yong, 2024). To address these challenges, national phage libraries have been established internationally (such as Belgium’s National Phage Library and the U.S. IPATH) to promote standardized procedures. Domestically, consensus guidelines are being developed to define protocols for phage isolation, identification, preparation, storage, and clinical application, aiming to achieve scalable and accessible phage therapy (Rochegüe et al., 2021). Overall, with the accumulation of clinical data, the development of engineered phages, and the refinement of regulatory frameworks, phage therapy is transitioning from the laboratory to the clinic, emerging as a promising innovative treatment for combating multidrug-resistant bacterial infections.

## 4 Clinical translation pathway: from mechanism to application

### 4.1 Biomarker development: identifying beneficiary populations

The primary challenge in translating microbiome-mediated radiosensitivity into clinical practice lies in developing quantifiable, reproducible biomarkers to achieve “precision prediction and precision intervention.” Based on their origin and function, these biomarkers can be categorized into the following three types:

#### 4.1.1 Microbiome biomarkers

Microbial community structure, characterized by 16S rRNA sequencing or metagenomic sequencing, serves as a potential marker for predicting radiotherapy efficacy and toxicity (Liang et al., 2025; Liu et al., 2021). For instance, patients with higher gut bifidobacteria abundance often exhibit better response to radiotherapy and lower incidence of radiation enteritis (Zhang et al., 2019; Häfner and Debus, 2016). Furthermore, the increased abundance of Actinobacteria and Veillonellaceae 6 months after radiotherapy in patients with nasopharyngeal carcinoma suggests delayed healing of oral mucositis, indicating that these microbes may serve as biomarkers for predicting the recovery speed of oral mucositis (Jiang et al., 2022).

#### 4.1.2 Metabolic biomarkers

The levels of beneficial metabolites in serum or feces directly reflect the functional state of the microbiota, making them more clinically useful markers (Bar et al., 2020; Zierer et al., 2018). For example, colorectal cancer patients with fecal butyrate concentrations >5 mmol/L may exhibit higher tumor regression rates during clinical radiotherapy, with statistically significant results (*p* = 0.01) (Park et al., 2020); In a prospective cohort study of cancer patients undergoing pelvic radiotherapy, researchers measured serum indole-3-propionic acid (IPA) concentrations before treatment. Serum IPA levels exceeding 2 μmol/L were significantly associated with reduced intestinal toxicity during radiotherapy, suggesting IPA may serve as a biomarker for radiotherapy toxicity risk or a potential therapeutic target. Propionate (IPA) levels before radiotherapy. Serum IPA concentrations exceeding 2 μmol/L were associated with a significant reduction in intestinal toxicity during radiotherapy, suggesting that IPA may serve as a biomarker for radiotherapy toxicity risk or a potential target for protective interventions (Xiao et al., 2020).

#### 4.1.3 Host response biomarkers

The microbiota influences radiosensitivity by regulating host immune and metabolic states. Therefore, host immune markers (e.g., peripheral blood Treg cell proportion, IL-10 concentration) or oxidative stress markers (e.g., serum MDA concentration, Nrf2 expression levels) can serve as co-markers. For instance, following whole-body 5 Gy *γ*-ray irradiation in mice, the survival rate of CD4^+^CD25^+^Foxp3^+^ Tregs in blood, lymph nodes, spleen, and thymus was significantly higher than that of CD4^+^CD25^−^ effector T cells. Radiation exposure significantly increased the survival rate of CD4^+^CD25^+^Foxp3^+^ Tregs in blood, lymph nodes, spleen, and thymus compared to CD4^+^CD25^−^ effector T cells. Tregs exhibited lower rates of apoptosis and higher Bcl-2 expression, indicating greater resistance to γ radiation (Elson and Cong, 2012; Gaweł et al., 2004; Zhou et al., 2020; Qu et al., 2010).

The primary challenges in current biomarker development include: (1) lack of standardized detection methods (e.g., variations in sequencing platforms and metabolite detection technologies); (2) interference from individual dietary habits, lifestyle factors, and underlying diseases; (3) most biomarkers remain based on small-sample studies, requiring large-scale prospective cohort validation. Future efforts should focus on establishing multi-center, standardized clinical studies to develop integrated biomarker panels linking “microbiota-metabolites-host responses,” thereby enhancing predictive accuracy.

### 4.2 Patient stratification: enabling precision radiotherapy

Based on the biomarkers above, cancer patients can be stratified according to radiation sensitivity and potential benefit from intervention, providing a basis for developing personalized radiotherapy plans. Taking colorectal cancer as an example, patients can be categorized into the following three groups:

#### 4.2.1 High-benefit cohort

Biomarker characteristics include “high abundance of beneficial bacteria, elevated short-chain fatty acids (SCFA)/indolepropionic acid (IPA) levels, and reduced proportion of regulatory T cells (Treg).” These patients exhibit high radiosensitivity and good tolerance to normal tissue. Probiotic intervention may be considered in combination with standard radiotherapy to enhance efficacy further (Zhang et al., 2019; Sánchez-Alcoholado et al., 2021; Kusumo et al., 2019). Concurrently, radiotherapy doses may be appropriately increased (e.g., a local boost to 60 Gy) to reduce the risk of local recurrence (Law, 2017).

#### 4.2.2 Low-benefit cohort

Biomarker characteristics include “low abundance of beneficial bacteria, reduced short-chain fatty acids (SCFA)/indolepropionic acid (IPA) levels, and elevated proportion of regulatory T cells (Tregs).” These patients exhibit low radiosensitivity and a high risk of normal tissue toxicity (Schaue and McBride, 2012; Wang et al., 2025). Prioritize microbiome remodeling interventions (e.g., fecal microbiota transplantation combined with antibiotic decontamination) before reassessing the feasibility of radiotherapy. Concurrently, consider combining immune checkpoint inhibitors (e.g., PD-1 antibodies) to reverse tumor immunosuppressive microenvironments and enhance the efficacy of radiotherapy. The key to patient stratification lies in dynamic monitoring: During radiotherapy (e.g., every 2 weeks), biomarkers must be repeatedly tested to detect dysbiosis or decreased metabolite levels promptly. Intervention strategies should then be adjusted (e.g., switching probiotic strains, increasing prebiotic doses) to prevent reduced efficacy or increased toxicity caused by dynamic changes in the microbiota.

### 4.3 Intervention window: timing for maximum efficacy

The timing window for targeted microbiome interventions directly influences their efficacy in modulating radiosensitivity and requires optimization based on radiotherapy protocols and the dynamic patterns of microbiome changes (Crawford and Gordon, 2005). Integrating preclinical research and early clinical evidence, the recommended intervention time windows are as follows:

#### 4.3.1 Intervention window before radiotherapy (pretreatment period)

It is recommended to initiate interventions (such as oral probiotics and prebiotics) 2–4 weeks before the commencement of radiotherapy. The goal is to establish a gut microbiota structure enriched with beneficial bacteria and elevate levels of beneficial metabolites. The core significance of this window lies in: (1) Allowing sufficient time for the microbiota to colonize and adapt metabolically, preventing radiation damage before the microbial community stabilizes after radiotherapy initiation; (2) Preemptively enhancing intestinal barrier function and host antioxidant capacity to reduce the risk of normal tissue injury during the early stages of radiotherapy. For example, a prospective study of cervical cancer patients demonstrated that daily oral administration of 1 × 10^10^ CFU of *L. acidophilus* and *B. bifidum* during radiotherapy significantly reduced the incidence of radiation-induced diarrhea (≥Grade 2) (control group, 45% vs. probiotic group, 9%) (Chitapanarux et al., 2010).

#### 4.3.2 Intervention window during radiotherapy (maintenance phase)

Continuous intervention is required throughout radiotherapy to maintain stable microbiota structure and beneficial metabolite levels. During this phase, dosage should be adjusted based on patient tolerance (e.g., temporarily reducing probiotic dosage to 5 × 10^9^ CFU/day if abdominal distension or diarrhea occurs) and combined with symptomatic treatment (e.g., using montmorillonite powder to protect the intestinal mucosa) (Penet et al., 2021) 0.85% of research studies demonstrated that probiotics significantly reduced the incidence of radiation/chemotherapy-associated diarrhea. Most trials showed that the Shannon index decreased less in the probiotic group than in the control group (statistically significant), suggesting that probiotics have a protective effect on maintaining microbial diversity (Rodriguez-Arrastia et al., 2021).

#### 4.3.3 Post-radiotherapy intervention window (recovery phase)

Continued intervention is necessary after radiotherapy concludes to facilitate gut microbiota restoration and promote normal tissue repair. During this phase, prebiotic doses may be gradually increased to stimulate the proliferation of intestinal anaerobic bacteria and accelerate the recovery of the microbial community structure (Moraitis et al., 2023). In a randomized controlled trial, 74 cervical cancer patients were divided into an observation group (*n* = 40) and a control group (*n* = 34). The observation group received oral probiotics (420 mg per dose, three times daily) starting 7 days before radiotherapy and continuing for 8 weeks after treatment completion; the control group received only conventional three-dimensional conformal radiotherapy. Results showed that the incidence of acute radiation enteritis in the observation group was 47.5%, significantly lower than the 70.6% in the control group (*p* < 0.05). This study suggests that a probiotic intervention during and after radiotherapy can effectively reduce the risk of acute radiation enteritis (Chen et al., 2014).

It should be noted that radiotherapy regimens (such as fractionated doses and total course duration) vary across tumor types, necessitating corresponding adjustments to the intervention time window. For instance, SBRT (stereotactic body radiation therapy), with its short course (typically 1–5 fractions), requires initiating high-intensity intervention 1 week before treatment to rapidly modulate the microbial state (Feigenberg et al., 2025). In contrast, conventional fractionated radiotherapy (e.g., 25–30 fractions) may adopt a three-phase intervention strategy: “pretreatment – maintenance – recovery.”

## 5 Combined therapy strategy: synergistically enhancing radiotherapy

### 5.1 Combination with advanced radiotherapy techniques

#### 5.1.1 SBRT

SBRT rapidly kills tumor cells through a single or a few high-dose radiation treatments (e.g., 3 × 18 Gy). However, high-dose radiation may lead to the accumulation of immunosuppressive cells (e.g., M2 macrophages) in the tumor microenvironment, potentially affecting long-term efficacy (Swamy, 2023). At the same time, SBRT still carries a relatively high risk of radiation damage to adjacent normal tissues, such as the lungs and intestines (Heijmen, 2007). Microbiome interventions (such as probiotics combined with prebiotics) may synergize with SBRT through the following mechanisms: (1) Enhancing radiation-induced immunogenic cell death (ICD), promoting dendritic cell (DC) maturation and CTL infiltration, while reducing the proportion of immunosuppressive cells (Kolahi Sadeghi et al., 2024); (2) Activating the Nrf2 pathway via short-chain fatty acids (SCFAs), thereby enhancing antioxidant capacity in normal tissues and reducing the risk of SBRT-associated pulmonary fibrosis and intestinal perforation (Yarahmadi and Afkhami, 2023). For example, this multicenter randomized controlled trial targeted patients with early-stage inoperable non-small cell lung cancer. The intervention protocol involved initiating *Bifidobacterium lactis* (approximately 1 × 10^10^ CFU/day) 1 week before radiotherapy, continuing administration throughout the entire SBRT course, and extending treatment for an additional 2 weeks post-radiotherapy. Results demonstrated significantly enhanced activation of CD8^+^ T cells and dendritic cells (DCs) in the probiotic group, with approximately 30–40% reduction in grade 3 or higher pulmonary and intestinal radiotoxicity to 40%. Furthermore, the 1-year local control rate increased from 78% in the control group to 92%, indicating that probiotics enhance the effects of immunogenic cell death, protect normal tissues, and significantly improve local control rates (Lu et al., 2024).

##### 5.1.1.1 Clinical application recommendations

For patients with early-stage non-small cell lung cancer, liver cancer, and other conditions suitable for SBRT, initiate probiotic intervention (e.g., *Bifidobacterium lactis* 1 × 10^10^ CFU/day) 1 week before radiotherapy. Continue intervention throughout radiotherapy and for 2 weeks post-treatment. Simultaneously, monitor fecal short-chain fatty acid (SCFA) levels to ensure that the butyrate concentration remains above 5 mmol/L, thereby maximizing the synergistic effects.

#### 5.1.2 Proton therapy

Proton therapy precisely deposits radiation energy within tumor tissue through the Bragg peak effect, significantly reducing doses to normal tissues (e.g., reducing parotid gland doses by 30–50% in head and neck tumor proton therapy). However, it still cannot completely prevent radiation damage to areas such as the intestines and oral cavity (Gordon et al., 2021). The core objective of combining microbiome intervention with proton radiotherapy is to protect normal tissues further and enhance patient tolerance: (1) For head and neck cancer patients, oral probiotics (e.g., *Lactobacillus salivarius*) can reduce the incidence and severity of oral mucositis while maintaining oral microbiota balance (Peng et al., 2024); (2) For patients with pelvic tumors (e.g., cervical cancer, prostate cancer), probiotic intervention can reduce the risk of proton radiotherapy-associated gut microbiota translocation and urogenital infections (Wedlake, 2018).

##### 5.1.2.1 Clinical application recommendations

Patients with head and neck tumors should begin oral administration of *Lactobacillus salivarius* 2 weeks before initiating proton radiotherapy, supplemented with oral care (such as rinsing with probiotic solutions) (Peng et al., 2024); Pelvic tumor patients should take a combination of live strains *Lactobacillus acidophilus* and *Bifidobacterium bifidum*, approximately 5 × 10^9^ CFU daily, starting 2 weeks before radiotherapy and continuing throughout treatment until 2 weeks after radiotherapy completion (Chitapanarux et al., 2010).

### 5.2 Combination with immunotherapy/targeted therapy

#### 5.2.1 Immune checkpoint inhibitors (ICI)

ICI (such as PD-1/PD-L1 antibodies) enhance antitumor immune responses by releasing immune suppression of tumor cells against CTLs (Wu et al., 2022). However, their monotherapy efficacy is limited by the immunogenicity of the tumor microenvironment (Yazdani et al., 2021). Microbiome intervention can form a “triple synergy” with ICI and radiotherapy through the following mechanisms:

##### 5.2.1.1 Enhancing ICI efficacy

Probiotics (such as *Bifidobacterium* and *Akkermansia* bacteria) can reverse the immunosuppressive state of the tumor microenvironment by increasing SCFA levels in the gut, promoting DC cell maturation and Th1 cell differentiation, and reducing Treg cell and M2 macrophage infiltration, thereby enhancing ICI response rates (Velikova et al., 2021). For example, in animal models, oral administration of *Bifidobacterium* alone enhances tumor control, while combination with anti-PD-L1 nearly completely suppresses tumor growth. Following high-dose local radiotherapy, PD-L1 expression is upregulated in the tumor microenvironment; concurrent use of anti-PD-L1 significantly enhances the antitumor effects of radiotherapy. In practical clinical or research settings, the following two experimental approaches may be referenced: (1) Oral or topical administration of probiotics (e.g., *Bifidobacterium longum* or *Akkermansia muciniphila*); (2) Concurrent or sequential administration of radiotherapy (SBRT or high-dose fractionation) with PD-1/PD-L1 antibodies, followed by assessment of tumor volume, immune cell infiltration, and fecal SCFA levels to validate the synergistic effects of the triple combination.

##### 5.2.1.2 Reducing radiotherapy-related immune toxicity

Radiotherapy may induce gut microbiota translocation, activate systemic inflammatory responses, and increase the risk of ICI-associated colitis and pneumonia (Dubin et al., 2016); Probiotics can mitigate these effects by maintaining intestinal barrier integrity, reducing endotoxin entry into the bloodstream, and lowering levels of inflammatory cytokines such as IL-6 and TNF-*α* (Wu et al., 2022). Experimental data indicate that oral administration of *Lactobacillus rhamnosus* (LR) significantly reduces the severity of ICI-induced colitis (Li et al., 2021).

##### 5.2.1.3 Clinical application recommendations

For patients with advanced solid tumors (e.g., lung cancer, melanoma) scheduled to undergo “radiotherapy + ICI” therapy, initiate oral administration of a probiotic combination (e.g., *Bifidobacterium* + *Akkermansia* bacteria, total dose 1 × 10^10^ CFU/day) 2 weeks before treatment initiation. Continue intervention throughout therapy until 4 weeks after completion of the ICI course. Simultaneously monitor fecal microbiota composition and serum levels of inflammatory cytokines. If dysbiosis occurs (e.g., Proteobacteria phylum abundance >30%), promptly adjust the probiotic strains (Yang et al., 2017; Yoshimoto et al., 2020; Tassopoulos et al., 2017).

#### 5.2.2 Targeted therapies

Targeted therapies (such as anti-angiogenic drugs and EGFR inhibitors) inhibit tumor progression by specifically blocking tumor growth signals or angiogenesis, but they are prone to developing resistance and may not sufficiently sensitize tumors to radiotherapy (Chen et al., 2015). Microbiome interventions can enhance the combined effects of targeted therapies and radiotherapy through metabolic regulation and immune synergy:

Anti-angiogenic agents, such as bevacizumab, can improve hypoxia in the tumor microenvironment and enhance radiosensitivity; however, they may cause intestinal mucosal ischemia and exacerbate bacterial translocation (Yoshimoto et al., 2020; Tassopoulos et al., 2017). Probiotics (e.g., *Lactobacillus acidophilus*) promote intestinal mucosal vascular repair by secreting vascular endothelial growth factor (VEGF)-like substances (ElÇİ et al., 2022), while simultaneously inhibiting abnormal tumor vascular proliferation through short-chain fatty acids (SCFAs), thereby enhancing the efficacy of anti-angiogenic drugs (Gong et al., 2024).

EGFR inhibitors such as cetuximab are commonly used in the treatment of head and neck tumors and colorectal cancer. Still, their skin toxicity (e.g., rash) and intestinal toxicity may affect patient tolerance (Adams et al., 2009). Probiotics can enhance intestinal barrier function, mitigate drug-induced intestinal inflammation, and facilitate the smooth progression of radiotherapy (Wu et al., 2022).

## 6 Recommendations for trials design: standardization and biomarker integration

Rigorous trial design is essential for clinical translation, integrating microbiome biomarkers throughout.

### 6.1 Endpoint setting: a multi-level evaluation system

#### 6.1.1 Primary endpoint

##### 6.1.1.1 Therapeutic

Selected based on tumor type, such as objective response rate (ORR) for solid tumors, pathological complete response rate (pCR) after neoadjuvant therapy for rectal cancer, or progression-free survival (PFS) (Wong et al., 2012; Hua et al., 2022).

##### 6.1.1.2 Toxicity

For studies targeting the reduction of adverse effects, the incidence of ≥Grade 2 specific radiotherapy-related toxicities (e.g., radiation enteritis, oral mucositis) may be set as the primary endpoint (Vinogradskiy et al., 2018).

##### 6.1.1.3 Microbiome regulation endpoints

To ensure the intervention itself is effective, biological endpoints should be established, such as achieving a predetermined threshold in the abundance of specific beneficial bacteria (e.g., *Bifidobacterium*) post-intervention, or a significant increase in key metabolite levels (e.g., fecal butyrate).

#### 6.1.2 Secondary endpoints

##### 6.1.2.1 Immune microenvironment indicators

Dynamic changes in the ratio of cytotoxic T cells (CTL) to regulatory T cells (Treg) in peripheral blood, and alterations in serum cytokine levels (e.g., IFN-*γ*, IL-10) (Mahnke et al., 2007).

##### 6.1.2.2 Quality of life

Assessed using standardized scales such as the EORTC QLQ-C30 (Gundy et al., 2012).

##### 6.1.2.3 Long-term outcomes

Overall survival (OS), local control rate, and distant metastasis rate.

#### 6.1.3 Safety endpoints

Closely monitor the incidence of probiotic-related adverse events (e.g., bloating, allergies) and serious adverse events (SAEs).

Example: A Phase II trial of “probiotics combined with neoadjuvant radiotherapy for rectal cancer” may set “pCR rate” and “incidence of ≥Grade 2 radiation enteropathy” as co-primary endpoints, with “doubling of gut *Bifidobacterium* abundance” and “increased peripheral blood CTL/Treg ratio” as key secondary endpoints.

### 6.2 Time management: dynamic monitoring throughout the entire treatment process

The microbiome is a dynamic system, and experimental design must account for its temporal nature.

#### 6.2.1 Baseline period (2–4 weeks before radiotherapy)

Collect patient stool, blood, and oral saliva samples for metagenomic sequencing of the microbiome, metabolite detection (SCFAs, IPA), and immune cell typing. Detailed records of dietary habits and medication history (particularly antibiotics) will be maintained to exclude confounding factors.

#### 6.2.2 Intervention period (initiated 1–2 weeks before radiotherapy and maintained throughout treatment)

Administer probiotics/prebiotics according to protocol. Collect stool samples biweekly to dynamically track changes in microbial community structure and metabolites, allowing for a timely assessment of the intervention’s biological effects. Evaluate toxicity weekly per CTCAE criteria and assess tumor response every 4–6 weeks via imaging (CT/MRI).

#### 6.2.3 Follow-up period (6 months to 2 years after radiotherapy completion)

Biological samples collected every 3 months to assess long-term stability of microbiota and metabolites; regular imaging and clinical follow-ups conducted to record survival and recurrence data.

Important notes: For short-course radiotherapy such as SBRT, the baseline period should be shortened to an highly brief cycle immediately preceding treatment. Increase the frequency of sample collection during the intervention period (e.g., once weekly) to capture rapid changes in the microbiome.

### 6.3 Dose exploration: from empirical to precision

The dose–response relationship of microbiome interventions remains unclear (Lee et al., 2015) and requires exploration through early-stage trials.

#### 6.3.1 Probiotic dosage

Employ a classic dose-escalation design (e.g., 3 + 3 model), starting with a lower dose (e.g., 5 × 10^9 CFU/day). Gradually increase the dose based on tolerability and the modulation of microbial community effects (e.g., colonization rate of target bacteria) to determine the recommended phase II dose (RP2D).

#### 6.3.2 Prebiotic dosage

When combined with prebiotics, the optimal dosage must be individually determined to avoid gastrointestinal discomfort (such as bloating or diarrhea) caused by high doses. Start with a low dose and gradually increase it.

#### 6.3.3 Dose adjustment strategy

Preset dose adjustment criteria. If certain prognostic target biomarkers fail to meet standards, the dose may be increased; if ≥Grade 2 gastrointestinal adverse reactions occur, the dose should be temporarily reduced or suspended.

Example: A Phase I trial of “Probiotics Combined with Lung Cancer SBRT” may be designed with three dose groups (low dose, medium dose, higher dose) to evaluate the microbial colonization rate and adverse reaction incidence in each group, thereby determining the recommended Phase II dose (RP2D).

### 6.4 Biomarker integration: enabling dynamic personalized treatment

The core to the trial’s success lies in deeply integrating biomarkers to form a closed-loop management system encompassing prediction, monitoring, and adjustment.

#### 6.4.1 Inclusion screening biomarkers (predictive)

Only patients likely to benefit should be enrolled, such as those with beneficial bacterial abundance above the median at baseline, low harmful bacterial abundance, and no recent antibiotic use. This enhances the homogeneity of the trial population and improves response rates.

#### 6.4.2 Mid-term adjustment markers (monitoring)

At the midpoint of intervention (e.g., week 2 of radiotherapy), if fecal SCFA levels decrease rather than increase, or if target microbiota fail to colonize successfully, this indicates the current intervention protocol may be ineffective and should be promptly adjusted (e.g., by changing bacterial strains or increasing prebiotics).

#### 6.4.3 Therapeutic response predictors (evaluation)

Following radiotherapy, patients who exhibit sustained favorable immune changes—such as increased CTL ratios and decreased Treg ratios—demonstrate predictive indicators of a favorable long-term prognosis.

#### 6.4.4 Technical implementation

To meet the timeliness requirements of dynamic monitoring, rapid quantitative analysis of key microbial communities via qPCR is recommended, with targeted detection of metabolite levels using high-performance liquid chromatography-mass spectrometry (HPLC/MS).

## 7 Critical evaluation and discussion: confronting limitations and charting new paths

Despite the promising prospects of the microbiome in regulating radiosensitivity, it is essential to maintain a clear-eyed awareness of the significant challenges and controversies present in current research—this is crucial for advancing the field toward maturity.

### 7.1 Translational gap: challenges in bridging animal models to clinical practice

The primary reasons for the poor success rate in translating preclinical research findings into clinical applications are:

#### 7.1.1 Species differences

The gut microbiota composition of commonly used animal models (e.g., SPF-grade mice) differs significantly from that of humans [e.g., Bacteroidetes phylum abundance is approximately 10% in mice (Ley et al., 2005) versus 30–40% in humans (Turnbaugh et al., 2006)], and lacks human-specific bacterial groups (e.g., *Akkermansia* species) (Routy et al., 2018). Consequently, probiotic strains effective in animal studies (e.g., *Lactobacillus rhamnosus* GG) exhibit inconsistent efficacy in human trials (some studies report a 20% improvement in ORR, while others show no significant difference) (Anhê et al., 2015).

#### 7.1.2 Mismatch in radiotherapy protocols

Animal studies often employ high-dose single-fraction radiotherapy (e.g., 20 Gy), whereas human clinical trials predominantly use conventional fractionated radiotherapy (e.g., 2 Gy per fraction, 25 fractions total). The extent of radiation damage to the microbiota and its recovery patterns differ between species (animal microbiota recovers within 1 week, while human microbiota requires 4–8 weeks), leading to discrepancies in recommended intervention time windows (Zhao et al., 2021; Nam et al., 2013).

#### 7.1.3 Host heterogeneity

Mice are inbred strains with uniform genetic backgrounds, whereas humans exhibit individual genetic variations (e.g., TLR4 gene polymorphisms), leading to differing responses to microbiota metabolites (e.g., patients carrying the TLR4 rs4986790 allele exhibit reduced cellular responses to inflammatory stimuli) (Ferwerda et al., 2008; Barrodia et al., 2024).

Solution pathway: Employ the “Humanized Microbiome Animal Model (HMA)” by transplanting human fecal microbiota into germ-free mice to mimic human microbiome characteristics. Concurrently, conduct “preclinical-clinical” bridging studies using radiation therapy regimens and intervention time windows consistent with human protocols in animal experiments to enhance translational efficiency.

### 7.2 Research heterogeneity: the root cause of inconsistent findings

The association between the microbiome and radiosensitivity varies significantly across different studies [e.g., some studies show a positive correlation between *Bifidobacterium* and ORR, while others find no association (Wu et al., 2022; Sivan et al., 2015)]. This variability primarily stems from the following factors:

#### 7.2.1 Population and sample size

Most studies are single-center and small-sample (*n* < 50), with patients exhibiting diverse tumor types, stages, and underlying conditions (e.g., diabetes, inflammatory bowel disease), leading to significant variations in microbial profiles. For instance, the abundance of butyrate-producing bacteria in the intestines of diabetic patients is markedly reduced (Qin et al., 2012), potentially masking the effects of probiotic interventions.

Strong confounding factors—diet (e.g., high-fiber diets may increase SCFA levels) (Then et al., 2024), antibiotic use (within the past month can reduce *α* diversity by 50%) (Abeles et al., 2016), and proton pump inhibitor (PPI) use (affecting gastric acid secretion and altering gut microbiota) (Imhann et al., 2016) all interfere with the association between the microbiome and radiosensitivity.

#### 7.2.2 Technical batch effects

Differences in microbial classification accuracy across sequencing platforms (e.g., Illumina MiSeq vs. Ion Torrent)—such as a 20% disparity in detection rates for low-abundance taxa (<1%) (Salipante et al., 2014) and variations in statistical methods (e.g., using Shannon index vs. Chao1 index for α-diversity calculations) introduce interpretive biases in results. For example, the Shannon index showed significantly higher values in the probiotic group compared to the placebo group (*p* = 0.036), whereas the Chao1 index revealed no significant difference (Labenz et al., 2023).

#### 7.2.3 Radiotherapy dose error

Calibration discrepancies in radiotherapy dose delivery across different centers (e.g., ±5%) result in variations in the actual radiation doses received by tumors and normal tissues, thereby affecting the assessment of radiosensitivity (Cai et al., 2025). For example, when the same patient receives “50 Gy/25 fractions” radiotherapy at different centers, the actual tumor dose may differ by 2.5 Gy—sufficient to alter the microbiome’s response to radiotherapy (e.g., a 30–50% reduction in butyrate-producing bacteria abundance) (Li et al., 2021).

Resolution path: Conduct multicenter, large-sample studies (*n* ≥ 200) employing uniform inclusion/exclusion criteria (e.g., dietary control, antibiotic use); utilize standardized sequencing platforms (e.g., Illumina NovaSeq) and statistical methods (e.g., QIIME2 for microbiota analysis); precisely control radiation doses via intensity-modulated radiotherapy (IMRT) to minimize dose variability.

### 7.3 Intervention risk: safety is never a given

Microbiome-targeted interventions (such as probiotics and fecal microbiota transplantation) are generally safe, but potential risks still exist and require attention:

#### 7.3.1 Opportunistic infections

Probiotic use in immunocompromised patients (e.g., those with post-radiotherapy neutrophil counts <1 × 10^9^/L) may trigger adverse reactions (Khorashadizadeh et al., 2024). For instance, a survey of 499 cancer patients undergoing chemotherapy revealed that approximately 28% had used probiotics; concurrently, the study documented several cases of probiotic-associated lactobacillosis and fungemia (Ciernikova et al., 2017). These findings suggest that caution is warranted when using probiotics in patients with hematologic immunosuppression.

#### 7.3.2 Microbiome imbalance

Improper use of probiotics (such as long-term single-strain interventions) may lead to microbiome imbalance and inhibit the growth of beneficial bacteria (Nabavi-Rad et al., 2022).

#### 7.3.3 Metabolic side effects

Prebiotics (such as fructooligosaccharides) ferment in the gut, producing large amounts of gas that may trigger symptoms of bloating and abdominal pain in patients with irritable bowel syndrome (IBS) (Lian et al., 2022). Additionally, excessive short-chain fatty acids (SCFAs)—such as butyrate concentrations exceeding 8 mmol/L—may inhibit intestinal epithelial cell proliferation and impair mucosal repair (Zhi et al., 2021).

#### 7.3.4 Risks of fecal microbiota transplantation (FMT)

FMT may transmit pathogens carried by the donor (e.g., *Clostridium difficile*, viruses) or result in transplant failure due to donor-recipient microbiota mismatch (success rate approximately 60–70%) (Porcari et al., 2023).

Resolution pathway: For immunocompromised patients, administer low-dose probiotics (<5 × 10^9^ CFU/day) while monitoring complete blood counts; avoid long-term monostrain interventions and recommend multi-strain probiotics (≥3 strains) (Babot et al., 2018); conduct rigorous donor screening before FMT (e.g., viral testing, fecal pathogen culture) and employ “autologous fecal microbiota transplantation” to reduce rejection risks (Fa-Ming et al., 2017).

### 7.4 Technical bottlenecks: barriers limiting cognitive depth

Current technical approaches in microbiome research still have limitations that constrain mechanism elucidation and clinical application:

#### 7.4.1 Insufficient identification depth

16S rRNA sequencing can only identify species at the genus level, failing to distinguish between species or strains (Johnson et al., 2019) [e.g., *Bifidobacterium longum* and *Bifidobacterium breve* within the *Bifidobacterium* genus exhibit different regulatory effects on radiation sensitivity (Yifan et al., 2001)], thereby preventing precise localization of functional strains. While metagenomic sequencing can achieve species-level identification, its high cost limits its applicability in large-scale studies.

#### 7.4.2 Incomplete metabolite coverage

Existing studies predominantly focus on a limited number of metabolites, such as SCFAs and IPAs (Tian et al., 2020), while insufficient detection of other key metabolites (e.g., polyamines, indole sulfates) (Shi and Liu, 2022; Al-Dajani et al., 2024) may overlook important regulatory mechanisms [e.g., polyamines promote DNA repair in tumor cells and reduce radiosensitivity (Wu et al., 2025)].

#### 7.4.3 Establishing causality is challenging

Most studies employ correlation analyses (e.g., linking microbiota abundance to ORR), and observational research cannot establish a causal chain from “microbiota → metabolites → radiosensitivity.” While germ-free mouse models can validate findings, a translation gap persists, as previously noted.

#### 7.4.4 Lack of dynamic monitoring technology

Current microbiota detection methods require collecting fecal samples, making real-time dynamic monitoring impossible (e.g., daily monitoring of microbiota changes during radiotherapy). Meanwhile, microbial metabolites in blood (such as serum butyrate) have a short half-life (approximately 2 h), making it challenging to reflect the long-term functional status of the microbiota.

Solution path: Develop “low-cost metagenomic sequencing technology” to achieve species-level identification; employ “non-targeted metabolomics” (e.g., LC–MS/MS) to cover a broader range of metabolites; establish causal relationships by integrating “fecal microbiota transplantation with gene knockout mouse models.”

## 8 Conclusion

The microbiome has emerged as a key determinant of radiosensitivity by regulating core mechanisms including DNA damage repair, immune responses, metabolic reprogramming, and tumor microenvironment remodeling. Extensive preclinical evidence demonstrates its immense potential as a radiosensitizer and radiation protector for normal tissues, opening entirely new dimensions in cancer therapy. However, individual heterogeneity, unclear causality, the translational gap between animal models and clinical settings, and questions regarding the optimal strains, dosages, timing, and safety of intervention strategies remain major obstacles to achieving clinical translation.

To overcome these bottlenecks, future research should focus on:

### 8.1 Deepening mechanism research and biomarker discovery

Systematically elucidate the molecular pathways of key bacterial strains and their metabolites (such as butyrate) in specific tumor and radiotherapy contexts, constructing a microbiome fingerprint capable of predicting treatment efficacy and toxicity.

### 8.2 Exploring synergistic effects with advanced radiotherapy technologies

Investigating the response patterns of the microbiome to emerging techniques such as stereotactic radiotherapy and proton therapy, and developing optimal “technology-microbiome” combination strategies.

### 8.3 Developing precision-engineered intervention strategies

Moving beyond traditional probiotics, we are pioneering innovative approaches such as phage-mediated clearance, synthetic microbial communities, and engineered probiotics to achieve targeted modulation of the tumor microenvironment.

### 8.4 Expanding the frontiers of combination therapies

Conducting systematic evaluations of synergistic effects between the microbiome and immune checkpoint inhibitors, targeted therapies, and other agents to clarify their role in reshaping the tumor immune microenvironment.

In summary, microbiome research is propelling radiotherapy from a purely physical technique into a new era of precision medicine deeply integrated with host biology. Although the path ahead is fraught with challenges, through interdisciplinary collaboration, targeting the microbiome will undoubtedly yield breakthroughs in enhancing radiotherapy efficacy and improving patient quality of life.
