# Supplementary material for: Microbiota-mediated modulation of radiosensitivity: mechanisms and therapeutic prospects of oral and gut microbiota, metabolites, and probiotics
Source: Front Microbiol. 2025 Dec 10;16:1689735. doi: 10.3389/fmicb.2025.1689735 (PMC12728358; doi:10.3389/fmicb.2025.1689735)
Supplement: Supplementary file 1 [file Table_1.DOCX]

**Table 1: Evidence Stratification of Microbiome-Related Factors and Interventions**

| **Evidence Level** | **Study Type** | **Core Feature** | **Typical Cases** |
| --- | --- | --- | --- |
| Grade I | Small-scale clinical trial | Sample size: 10–100 cases, primarily prospective design, including healthy controls or stratified by treatment outcomes. | Radiotherapy for Liver Cancer: A study of 24 patients revealed significantly higher levels of bacterial c-di-AMP in the stool of the radiotherapy-responsive group, which enhanced therapeutic efficacy by activating the cGAS-STING-IFN-I pathway^[1]^. |
|  |  |  | Radiotherapy and Chemotherapy for Lung Cancer: A small-scale study indicates that increased Akkerbacterium abundance following radiotherapy and chemotherapy correlates with longer distant metastasis-free survival in patients^[2]^. |
| Grade II | Animal models, organoid research | Using organoids derived from model organisms (primarily mice) or patient sources, mechanism elucidation and intervention validation can be performed. | Radiation-induced rectal disease: Mouse models confirm that radiation exposure reduces Akkermansia bacteria, and supplementation with Akkermansia mitigates intestinal damage by elevating the metabolite 3-hydroxybutyrate and downregulating the GPR43-IL6 pathway^[3]^. |
| Grade III | In vitro cell experiments | Exploration of Mechanisms at the Cellular Level | Immune Cell Regulation: In vitro experiments reveal that the phospholipid component a15:0-i15:0 PE from Akk bacteria can regulate dendritic cell activation via TLR2-TLR1 receptors^[4, 5]^. |

**References**

1. Li, Z., et al., *Gut microbiota modulate radiotherapy-associated antitumor immune responses against hepatocellular carcinoma Via STING signaling.* Gut Microbes, 2022. **14**(1): p. 2119055.

2. Wu, L., et al., *Gut microbiota predictive of the efficacy of consolidation immunotherapy and chemoradiotherapy toxicity in lung cancer.* Med, 2025: p. 100877.

3. Ge, Z., et al., *Gut Microbiota-Derived 3-Hydroxybutyrate Blocks GPR43-Mediated IL6 Signaling to Ameliorate Radiation Proctopathy.* Adv Sci (Weinh), 2024. **11**(28): p. e2306217.

4. Brown, E.M., J. Clardy, and R.J. Xavier, *Gut microbiome lipid metabolism and its impact on host physiology.* Cell Host Microbe, 2023. **31**(2): p. 173-186.

5. Ryan, E., et al., *Lipidomic Analysis Reveals Differences in Bacteroides Species Driven Largely by Plasmalogens, Glycerophosphoinositols and Certain Sphingolipids.* Metabolites, 2023. **13**(3).
