# Supplementary material for: Microbiota-mediated modulation of radiosensitivity: mechanisms and therapeutic prospects of oral and gut microbiota, metabolites, and probiotics
Source: Front Microbiol. 2025 Dec 10;16:1689735. doi: 10.3389/fmicb.2025.1689735 (PMC12728358; doi:10.3389/fmicb.2025.1689735)
Supplement: Supplementary file 2 [file Table_2.DOCX]

**Table 2: Comparison of Physiological Functions Between Traditional Probiotics and Next-Generation Probiotics (NGPs)**

| **Functional Dimension** | **Traditional Probiotics** | **Next-Generation Probiotics (NGPs)** |
| --- | --- | --- |
| SCFA Production | Primarily lactate; low and limited SCFA yield^[1]^ | Highly efficient production of butyrate, propionate, and acetate; complex metabolic networks directly involved in host metabolic regulation. These metabolites enhance intestinal barrier function, modulate immune responses, and improve metabolism^[1, 2]^ |
| Immune Regulation | Indirect anti-inflammatory effects; lack disease specificity^[1]^ | Directly modulates immune cell activity (e.g., Tregs, dendritic cells, B cells, macrophages), precisely regulates inflammatory pathways, reduces inflammatory responses while enhancing immune defense, applicable to cancer and autoimmune diseases ^[1, 3]^ |
| Intestinal Barrier Protection | Dependent on microbial competition, with limited efficacy^[1]^ | Directly repairs the mucus layer and tight junction proteins, significantly enhancing physical and immune barriers^[1, 3, 4]^ |
| Disease Intervention Scope | Primarily used for common intestinal disorders like diarrhea and indigestion^[1]^ | Potential applications in metabolic disorders (e.g., obesity, type 2 diabetes, non-alcoholic fatty liver disease), inflammatory bowel diseases (e.g., Crohn's disease, ulcerative colitis), cancers (e.g., colorectal, gastric, hepatocellular carcinoma), cardiovascular diseases (e.g., atherosclerosis, hypertension), and neurological disorders (e.g., depression, anxiety, Alzheimer's disease)^[3]^ |

**References**

1. Loo, K.-Y., et al., *A Current Overview of Next-Generation Probiotics and Their Prospects in Health and Disease Management.* Progress In Microbes & Molecular Biology, 2024. **7**(1).

2. Huang, Y., et al., *Role of Gut Microecology in the Pathogenesis of Drug-Induced Liver Injury and Emerging Therapeutic Strategies.* Molecules, 2024. **29**(11): p. 2663.

3. Al-Fakhrany, O.M. and E. Elekhnawy, *Next-generation probiotics: the upcoming biotherapeutics.* Mol Biol Rep, 2024. **51**(1): p. 505.

4. Mengling, Y., et al., *Research progress of intestinal barrier and gut microbiota in*

*ulcerative colitis.* Microbiology China, 2024. **51**(09): p. 3384-3397.
